# The Book World of Medicine and Science

**Published:** 1894-08-25

**Authors:** 


### THE BOOK WORLD OF MEDICINE AND SCIENCE.

BEGINNERS' GUIDE TO PHOTOGRAPHY, SHOWING HOW TO BUY A CAMERA AND HOW TO USE IT. By A FELLOW OF THE CHEMICAL SOCIETY. (Published by Perken, Son, and Rayment. Price 6d.)

Several of the leading photographic firms now issue a kind of "instructional" catalogue of their wares, and the little book before us belongs in some measure to this class. This fact, however, so far from diminishing, rather adds to its value, so long as it is a question of dealing with a first-rate firm, and in the present volume the tyro will find an admirable and practical introduction to his art, and many useful suggestions besides as to necessary or desirable equipments. Of course, there is no pretence made of doing more than merely guiding the beginner through his first steps, but it will be his own fault if he is unable to obtain excellent results in the practice of picture-making. The section on photo-micrography strikes us as perhaps the one most open to criticism, but most persons will probably not attempt this more difficult work until they have already gained sufficient experience in the simpler operations, such at least as will enable them to consult larger treatises with advantage. For all the ordinary work of the photographer we can cordially and unhesitatingly recommend this little work, which is equally admirable, both as regards its practical character and in its small cost.

ANALYSES OF TWELVE THOUSAND PRESCRIPTIONS. Compiled by W. MARTINDALE, F.C.S. (H. K. Lewis, Gower Street, London. 1894.)

The object of the author is to indicate to the profession the tendency of modern therapeutics, and to call attention to certain anomalies in construction of the British Pharmacopœia. Thus, from an analysis of 12,000 prescriptions dispensed in six pharmacies in typical centres of the United Kingdom, namely, at Aberdeen, Bournemouth, Carlisle, Cork, Oxford, and London, Mr. Martindale has been able to prove that many unofficial preparations have a most extensive demand, and certainly deserve a place in the next issue of the Pharmacopœia, while many officially recognised preparations find no mention in the author's records. The positive evidence collected by Mr. Martindale has undeniable value, and no doubt use will be made of his data in future editions of the Pharmacopœia. But no justifiable inference can be drawn from the negative evidence in reference to drugs whose names find no mention in the records of Mr. Martindale's collaborateurs. Fashion plays so large a part in the ordering of drugs, and practitioners so closely imitate the methods of their colleagues in the same town or district, that it is quite possible to find a large number of drugs which do useful service, say, in Edinburgh or York, and which are never prescribed in Cork, Oxford, or Bournemouth. In the table of the "greatest frequency" drawn up by the author the following facts are of interest: Spiritus chloroformi heads the list with 1,117 entries in 12,000 prescriptions, tinctura nucis vomicæ holds the second place with 991 entries, glycerine the third with 875, and bicarbonate of soda, the fourth with 807. Pil aloes et myrrh only appears 35 times, ichthyol only 18 times, lotio hydragyri perchloride 13 times, lotio acidi carbolici 12 times. Trochesci ferri redacti, oleum copaibæ, pil cambogiæ co. find no entry at all. Of unofficial preparation syropus ferri quininæ et strychninæ phosphatum was prescribed 173 times, syrup hypophosphitum composisus 143 times, nepenthe 107 times, bromidia 48 times, and piperazine 22 times.

